# Strange Case of a Degenerating Human Body

**Published:** 1896-01

**Authors:** 


STRANGE CASE OF A DEGENERATING HUMAN BODY.—A medical case of the greatest interest was presented to a clinic at the New York College of Dentistry last week, when Dr. F. D. Weise, one of the medical staff, introduced John M. Molanski, who is suffering from what is known in medical science as acromygatia. It is regarded as one of the rarest and most mysterious diseases to which human kind is subject.

Medical authorities differ as to the exact nature of the disease, some believing that it is not really a disease, but a form of physical atavism, or a tendency to return to the original species, or a reversion to the primary type of man.

Molanski is undergoing a bodily metamorphosis. His face is gradually being transformed from its natural appearance into a strong animal type, with protruding under jaw and overhanging brows, which a heavy beard and head of hair but partially conceal. His hands and feet are growing larger and larger, and are already taking on the appearance of those of a monkey. He suffers no personal inconvenience, and is daily engaged in his business of cracker manufacturer, at No. 23 Cherry street. His disease, if it may be so called, is not contagious, being only a state of retrogression. It is not more than nine years ago that acromygalia first made its appearance and was recognized. There are only twenty cases on record, and that of Molanski is the only one now known to be in existence.—*N. Y. Herald*, Nov. 12, '95.

